# The impact of sunlight exposure on brain structural markers in the UK Biobank

**DOI:** 10.1038/s41598-024-59633-z

**Published:** 2024-05-05

**Authors:** Huihui Li, Fusheng Cui, Tong Wang, Weijing Wang, Dongfeng Zhang

**Affiliations:** Department of Epidemiology and Health Statistics, Public Health College, Qingdao University, No. 308 Ningxia Road, QingdaoShandong Province, 266071 China

**Keywords:** Cognitive ageing, Cognitive neuroscience, Environmental sciences

## Abstract

Sunlight is closely intertwined with daily life. It remains unclear whether there are associations between sunlight exposure and brain structural markers. General linear regression analysis was used to compare the differences in brain structural markers among different sunlight exposure time groups. Stratification analyses were performed based on sex, age, and diseases (hypertension, stroke, diabetes). Restricted cubic spline was performed to examine the dose–response relationship between natural sunlight exposure and brain structural markers, with further stratification by season. A negative association of sunlight exposure time with brain structural markers was found in the upper tertile compared to the lower tertile. Prolonged natural sunlight exposure was associated with the volumes of total brain (*β*: − 0.051, *P* < 0.001), white matter *(β*: − 0.031, *P* = 0.023), gray matter (*β*: − 0.067, *P* < 0.001), and white matter hyperintensities (*β*: 0.059, *P* < 0.001). These associations were more pronounced in males and individuals under the age of 60. The results of the restricted cubic spline analysis showed a nonlinear relationship between sunlight exposure and brain structural markers, with the direction changing around 2 h of sunlight exposure. This study demonstrates that prolonged exposure to natural sunlight is associated with brain structural markers change.

## Introduction

Sunlight is closely associated with human health. Sunlight plays a crucial role in maintaining overall health by participating in multiple processes such as skin synthesis of vitamin D^1,2^ and regulating the circadian rhythm^3,4^. However, inappropriate exposure to ultraviolet (UV) radiation from sunlight can result in both acute and chronic health consequences, including skin cancer^5^, sunburn (erythema)^6^, immunosuppression^7^, DNA damage^8^, and more. The UV radiation has the potential to suppress cell-mediated immune function, leading to inflammatory responses^7^, while the inflammatory response is recognized as one of the risk factors for dementia^9^. Additionally, worsening air pollution has contributed to the thinning of the ozone layer, reducing its capacity to absorb UV radiation, which may result in increased UV exposure for individuals^10^. Besides, it has been shown that individuals with lighter skin tones are more susceptible to the effects of UV radiation^11^.

The brain can also be affected by sunlight. Brain function relies on the delivery of oxygen and nutrients through blood circulation and depends on the brain’s ability to maintain thermal balance. When exposed to sunlight, more blood flows away from the brain to regulate brain temperature, resulting in a reduced blood flow to the brain, which may lead to brain damage^12,13^. Besides, temperature is a key factor supporting the development of non-neuronal cells, leading to enlargement of the brain^14^. Studies have shown that brain hypothermia has a neuroprotective effect, and high temperature may increase brain temperature by altering several factors such as cerebral blood flow and blood temperature, thereby affecting brain function^15^. It has been reported that even the changes in brain temperature was less than 1 °C, it still can lead to functional changes in various regions of the nervous system, affecting nerve conduction velocity and synaptic transmission^16^. Experimental studies also have found that direct exposure of the head and neck to sunlight radiation can result in a core temperature increase of 1 °C, and may impair motor-cognitive functions^17^. Moreover, the nitric oxide (NO), acting as a endogenous vasodilator, can improve blood flow and lower blood pressure, thereby prompting brain health. It has been indicated that exposure to UV radiation in sunlight may lead to the release of NO from the skin into the bloodstream^18^. However, the association of sunlight with brain structure remains incompletely understood now.

Nevertheless, there remains a gap in the directive investigation of the associations between natural sunlight exposure and brain structure. Research indicates that changes in brain morphology, such as white matter integrity, may precede and potentially lead to declines in cognitive function^19^, and individual differences in cognitive function are partially explained by variations in brain structure^20^. White matter hyperintensity, as one of the brain structural markers, is associated with pathologies of Alzheimer's disease^21,22^. Therefore, brain structural markers, such as the volumes of total brain, white matter, gray matter, and white matter hyperintensities, can serve as good representative indicators for brain function.

In this study, we aimed to explore the relationships between sunlight exposure and brain structural markers using the data from the UK Biobank cohort. Furthermore, since season, sex, and age differences in the association between sunlight exposure and brain function (such as cognition)^2^, we further conducted stratified analyses based on these factors. In addition, considering that hypertension^23,24^, stroke^25,26^, and diabetes^27,28^ are closely associated with brain structure as well as cognitive impairment, we also tried to analyze the relationships between sunlight exposure and brain structure in these diseases groups, respectively.

## Results

A total of 27,474 participants (mean age 55.01 ± 7.57 years) who completed brain scan were included in baseline characterization analysis. (Table 1) Compared to the group with shorter sunlight exposure time (< = 1.5 h), the group with longer time tended to be older, more likely to consist of males, engage in high level of physical activities, and have appropriate sleep duration.

**Table 1:** The characteristics of participants grouped by sunlight exposure time at baseline.

	Overall (N = 27,474)	Sunlight exposure time		
		Tertile 1 < = 1.5 h	Tertile 2 1.5–3 h	Tertile 3 > 3 h
		(N = 11,043)	(N = 9847)	(N = 6584)
Age (mean (SD))	55.01 (7.57)	53.32 (7.15)	55.37 (7.57)	57.29 (7.60)
Sex (%)				
Female	13,694 (49.8)	5890 (53.3)	5064 (51.4)	2740 (41.6)
Male	13,780 (50.2)	5153 (46.7)	4783 (48.6)	3844 (58.4)
Sleep duration (%)				
7–8 h	7590 (27.6)	2965 (26.8)	2699 (27.4)	1926 (29.3)
< 7 or > 8 h	19,884 (72.4)	8078 (73.2)	7148 (72.6)	4658 (70.7)
Skin color (%)				
Very fair	2149 (7.8)	1067 (9.7)	675 (6.9)	407 (6.2)
Fair	18,957 (69.0)	7665 (69.4)	6846 (69.5)	4446 (67.5)
Light olive	5462 (19.9)	1995 (18.1)	1995 (20.3)	1472 (22.4)
Dark olive	419 (1.5)	132 (1.2)	147 (1.5)	140 (2.1)
Brown	431 (1.6)	166 (1.5)	157 (1.6)	108 (1.6)
Black	56 (0.2)	18 (0.2)	27 (0.3)	11 (0.2)
Use of sun/UV protection (%)				
Never/rarely	2060 (7.5)	803 (7.3)	696 (7.1)	561 (8.5)
Sometimes	9399 (34.2)	3728 (33.8)	3369 (34.2)	2302 (35.0)
Most of the time	10,720 (39.0)	4485 (40.6)	3874 (39.3)	2361 (35.9)
Always	5223 (19.0)	1976 (17.9)	1892 (19.2)	1355 (20.6)
Do not go out in sunshine	72 (0.3)	51 (0.5)	16 (0.2)	5 (0.1)
History of fractures in the past 5 years (%)				
No	25,291 (92.1)	10,232 (92.7)	9045 (91.9)	6014 (91.3)
Yes	2183 (7.9)	811 (7.3)	802 (8.1)	570 (8.7)
Smoke status (%)				
Never	16,637 (60.6)	7118 (64.5)	5900 (59.9)	3619 (55.0)
Previous	9125 (33.2)	3345 (30.3)	3306 (33.6)	2474 (37.6)
Current	1712 (6.2)	580 (5.3)	641 (6.5)	491 (7.5)
Alcohol status (%)				
Never	590 (2.1)	269 (2.4)	191 (1.9)	130 (2.0)
Previous	561 (2.0)	219 (2.0)	201 (2.0)	141 (2.1)
Current	26,323 (95.8)	10,555 (95.6)	9455 (96.0)	6313 (95.9)
BMI (mean (SD))	26.58 (4.18)	26.51 (4.35)	26.51 (4.10)	26.79 (3.98)
Physical activity (%)				
Low	5114 (18.6)	3113 (28.2)	1414 (14.4)	587 (8.9)
Moderate	11,606 (42.2)	4989 (45.2)	4361 (44.3)	2256 (34.3)
High	10,754 (39.1)	2941 (26.6)	4072 (41.4)	3741 (56.8)
PM2.5 (median [IQR])	9.86 [9.17, 10.53]	9.89 [9.23, 10.57]	9.84 [9.17, 10.52]	9.81 [9.10, 10.50]
TDI (median [IQR])	− 2.63 [− 3.88, − 0.55]	− 2.60 [− 3.90, − 0.49]	− 2.63 [− 3.88, − 0.54]	− 2.66 [− 3.84, − 0.68]
Years of education (%)				
10-years	5216 (19.0)	1503 (13.6)	1908 (19.4)	1805 (27.4)
13-years	1593 (5.8)	717 (6.5)	534 (5.4)	342 (5.2)
15-years	3388 (12.3)	1146 (10.4)	1311 (13.3)	931 (14.1)
19-years	4246 (15.5)	1426 (12.9)	1547 (15.7)	1273 (19.3)
20-years	13,031 (47.4)	6251 (56.6)	4547 (46.2)	2233 (33.9)
Employment status (%)				
No	8624 (31.4)	2010 (18.2)	3444 (35.0)	3170 (48.1)
Yes	18,850 (68.6)	9033 (81.8)	6403 (65.0)	3414 (51.9)
Vitamin D supplementation (%)				
No	26,292 (95.7)	10,554 (95.6)	9417 (95.6)	6321 (96.0)
Yes	1182 (4.3)	489 (4.4)	430 (4.4)	263 (4.0)
History of diabetes (%)				
No	26,608 (96.8)	10,716 (97.0)	9560 (97.1)	6332 (96.2)
Yes	866 (3.2)	327 (3.0)	287 (2.9)	252 (3.8)
History of hypertension (%)				
No	20,850 (75.9)	8650 (78.3)	7435 (75.5)	4765 (72.4)
Yes	6624 (24.1)	2393 (21.7)	2412 (24.5)	1819 (27.6)
History of stroke (%)				
No	27,112 (98.7)	10,939 (99.1)	9701 (98.5)	6472 (98.3)
Yes	362 (1.3)	104 (0.9)	146 (1.5)	112 (1.7)
PRS for Alzheimer’s disease (mean (SD))	0.03 (0.98)	0.03 (0.98)	0.04 (0.98)	0.04 (0.99)

### Main analysis

The results of associations between sunlight exposure time and brain structural markers were presented in Table 2. Comparing to Tertile 1, prolong natural sunlight exposure time (Tertile 3) was negatively associated with the volumes of total brain (*β*: − 0.051, *P* < 0.001), white matter (*β*: − 0.031, *P* = 0.023), gray matter (*β*: − 0.067, *P* < 0.001), and positively associated with white matter hyperintensities (*β*: 0.059, *P* < 0.001). Longer sunlight exposure time was negatively associated with smaller subcortical volumes of thalamus (*β*: − 0.060, *P* < 0.001), caudate (*β*: − 0.040, *P* = 0.012), putamen (*β*: − 0.031, *P* = 0.032), hippocampus (*β*: − 0.046, *P* = 0.003), and accumbens (*β*: − 0.041, *P* = 0.006). Similarly, prolonged sunlight exposure was associated with reduced gray matter volumes in the putamen (*β*: − 0.060, P < 0.001), hippocampus (*β*: − 0.043, *P* = 0.004), and amygdala (*β*: − 0.073, *P* < 0.001).

**Table 2:** Association between sunlight exposure time and brain structural markers.

Brain structural markers	Sunlight exposure time					
	Tertile 2^#^			Tertile 3^#^		
	*β*	SE	*P*	*β*	SE	*P*
Global measures						
Total brain volume	− 0.025	0.011	0.026	− 0.051	0.013	< 0.001
Volume of white matter	− 0.014	0.012	0.222	− 0.031	0.014	0.023
Volume of gray matter	− 0.035	0.011	0.002	− 0.067	0.014	< 0.001
Volume of white matter hyperintensities	0.013	0.013	0.314	0.059	0.016	< 0.001
Subcortical regions						
Volume of thalamus	− 0.029	0.012	0.014	− 0.060	0.014	< 0.001
Volume of caudate	− 0.030	0.013	0.025	− 0.040	0.016	0.012
Volume of putamen	− 0.022	0.012	0.065	− 0.031	0.014	0.032
Volume of pallidum	− 0.027	0.013	0.044	− 0.026	0.016	0.106
Volume of hippocampus	− 0.010	0.013	0.450	− 0.046	0.016	0.003
Volume of amygdala	− 0.003	0.014	0.819	− 0.031	0.016	0.054
Volume of accumbens	− 0.004	0.013	0.748	− 0.041	0.015	0.006
Regional gray matter volumes						
Volume of thalamus	− 0.007	0.014	0.613	− 0.022	0.016	0.179
Volume of caudate	− 0.010	0.014	0.461	0.009	0.016	0.566
Volume of putamen	− 0.052	0.014	0.000	− 0.060	0.017	< 0.001
Volume of pallidum	0.001	0.014	0.960	0.018	0.017	0.276
Volume of hippocampus	− 0.018	0.013	0.154	− 0.043	0.015	0.004
Volume of amygdala	− 0.038	0.012	0.002	− 0.070	0.015	< 0.001

^**#**^The tertile 1 of sunlight exposure time serving as reference.

### Stratified analysis

The male brain structure appears to be more susceptible to the effects of sunlight exposure compared to females. (Supplementary Table S3) Among males, we found that prolong sunlight exposure was negatively associated with total brain volume, gray matter volume, subcortical volumes of the thalamus and caudate, gray matter volumes of the putamen, hippocampus, and amygdala. It was also associated with an increase in the volume of white matter hyperintensity. In females, it was only associated with total brain volume, gray matter volume, subcortical volumes of the thalamus and hippocampus.

While comparing to the group aged 60 years and above, the group under 60 years showed a broader range of correlations between sunlight exposure and brain structural markers. (Supplementary Table S4) With longer sunlight exposure time, participants under 60 years exhibited shrinkage in volumes of total brain, white matter, gray matter, and increase in volume of white matter hyperintensities. However, only a correlation with gray matter volume was found in the population aged 60 years and above.

In the group of hypertension, prolong sunlight exposure time was negatively associated with total brain volume, gray matter volume, white matter volume, subcortical volumes in thalamus and hippocampus, and the gray matter volumes in putamen, hippocampus and amygdala. However, no significant associations were observed in the stroke and diabetes individuals. (Supplementary Table S5).

### Restricted cubic spline

**Figure 1:**
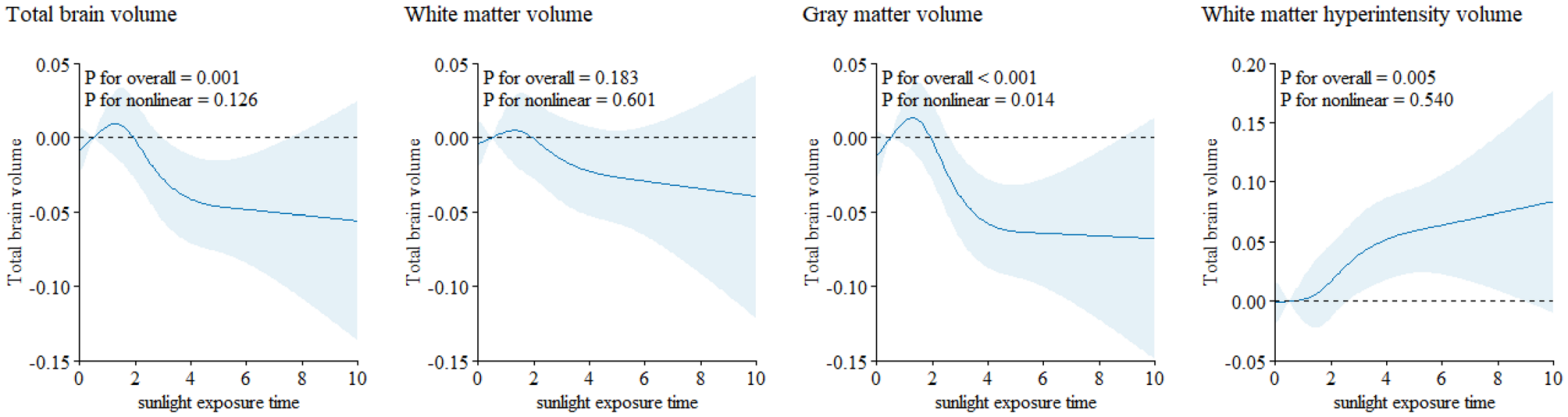
The restricted cubic splines of natural sunlight exposure with brain structure markers.

**Figure 2:**
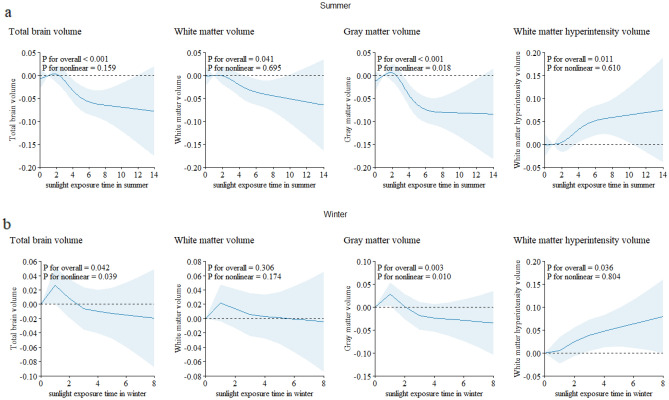
The restricted cubic splines of natural sunlight exposure with brain structure markers stratified by season. (**a**) Natural sunlight exposure in summer. (**b**) Natural sunlight exposure in winter.

The restricted cubic spline illustrated a nonlinear relationship between sunlight exposure duration and brain structural markers. Within 2 h of sunlight exposure, total brain volume, white matter volume, and gray matter volume increased as sunlight exposure time increasing. However, when daily sunlight exposure approximately exceeded 2 h, we observed a decrease in total brain volume, gray matter volume, white matter volume and volumes of certain subcortical regions with prolonged sunlight exposure duration (Fig. 1 and Supplementary Fig. S1) . When stratified by season, as sunlight exposure duration increasing, the total brain volume, white matter volume, and gray matter volume decreased more pronounced in the summer compared to winter (Fig. 2 and Supplementary Fig. S2). Regardless of the season, sunlight exposure time was associated with an increase in white matter hyperintensity volume.

### Sensitivity analysis

The sensitivity analyses results showed that our findings were robust. After excluding participants who developed dementia within the first 5 years and the first 10 years of follow-up, the results were still consistent with the main results. (Supplementary Tables S6 and S7) Similar results were also found between prolonged natural sunlight exposure and brain structural markers when baseline individuals with hypertension, diabetes, or stroke were further removed. (Supplementary Table S8) Results in the white population were also consistent with those of the primary analysis. (Supplementary Table S9) Prolong sunlight exposure time was associated with cognitive function tests. (Supplementary Table S10) In terms of cognitive function, as the duration of sunlight exposure increased, performance in visual declarative memory, working memory, verbal and numerical reasoning, processing speed, executive function, vocabulary, and non-verbal reasoning declined.

## Discussion

We observed that prolonged exposure to natural sunlight may be associated with adverse brain structure. This association varied in different age, sex, and season, and stronger negative correlations were found in males, those under 60 years old, and during the summer. Additionally, prolonged exposure to sunlight was correlated with cognitive decline. The restricted cubic spline results showed a non-linear association between sunlight exposure time and brain structural markers, with adverse changes after sunlight exposure time exceeding approximately 2 h.

Now the mechanisms by which prolong sunlight induces damage to brain structure are not fully understood. It may be as follows: (1) Sunlight increases brain temperature by affecting cerebral blood flow and blood temperature, and broad-spectrum light may also penetrate the skin and heat tissues, also increasing brain temperature^12,13,17^. Elevated brain temperature can alter resting potentials, action potentials, nerve conduction velocity, and synaptic transmission, leading to changes in brain function^16,29^. (2)The increase in brain temperature also affects the integrity of the blood–brain barrier, mitochondrial function, and decreases tolerance to potential insults to the brain^16^. (3) The UV radiation in natural sunlight can damage immune cells in the body, triggering inflammatory responses that can lead to damage^7,9^. (4) UV radiation in sunlight may induce systemic oxidative stress, which can affect the brain through mechanisms such as inflammation, cell apoptosis, and neuronal damage^30,31^.

We found that short-term exposure to sunlight (< 2 h) was associated with beneficial changes of brain structural markers. This may be due to the following reasons: (1) Most of the vitamin D in the body is synthesized from sunlight exposure to the skin^32^, and moderate sunlight exposure can maintain adequate levels of vitamin D which participates in maintaining brain function by regulating the expression of neurotrophic factors, the immune system and oxidative stress^1,33^; (2) Sunlight projects through atypical intrinsically photosensitive retinal ganglion cells (ipRGCs) to the suprachiasmatic nucleus (SCN) in the brain, regulating the circadian rhythm to maintain brain health^34,35^; (3) Exposure to sunlight can regulate the release of neurotransmitters such as dopamine and serotonin in the brain, contributing to brain health^36–38^.

The relationship between natural sunlight exposure and change in brain structure appeared to be more extensive in the summer season, in individuals younger than 60 years old, and males. This can be attributed to higher temperature and stronger UV radiation during the summer in the United Kingdom^39^. Additionally, during the summer, people tend to expose more skin due to warmer weather and clothing choices, leading to increased UV exposure. Younger individuals tend to engage in outdoor activities, and some of them may work outside, leading to prolonged sunlight exposure. Research has shown that among people over 20 years old, the frequency of sunburn decreases with age^40,41^. There are known structural and biological differences in the skin between sex^42,43^. Compared to females, males tend to be more sensitive to UV radiation and may experience immune-suppression reactions more frequently^44,45^. Conversely, the presence of estrogen in the female body may exert inhibitory effects on immune-suppression reactions^46^. Furthermore, males are generally less likely to use sun protection measures, resulting in greater sunlight exposure^41^.

The associations between sunlight exposure and brain structural marers are consistent with prospective studies in dementia populations. Ma et al. found a "J-shaped" relationship between sunlight exposure and the development of dementia, and we observed that high-dose sunlight exposure may have a damaging effect on brain structural markers^2^. The finding regarding the association of natural sunlight exposure with cognition align with previous comparative studies conducted on worker populations. Exposure to sunlight has been observed to decrease attention allocation and vigilance. Under both temperate and tropical climate conditions, sunlight exposure has been shown to result in cognitive impairment^47^. Dementia is a slowly progressive condition, and the cognition changes we focused on occur earlier than the diagnosis of dementia^48–51^. Besides, research indicates that the atrophy of white matter may lead to cognitive impairment such as vascular dementia and other related conditions^52–55^. Additionally, the atrophy of gray matter volume is also associated with the decline in cognition, such as in Alzheimer's disease^54,56^. Based on the association between natural sunlight and changes in brain structure, we hypothesize that brain structure may mediate the association between natural sunlight and cognition. However, further research is needed to confirm this hypothesis.

Our study has several strengths. First, this study represents the first exploration of the associations as well as the nonlinear relationship between natural sunlight exposure and brain structure in the general population. Second, we extensively adjusted for various potential confounding factors to control for influences from the environment, genetics, and other aspects. Furthermore, we conducted multi-level analyses stratified by season, age, sex, and four diseases to investigate variations among different subgroups.

However, there are still some limitations. First, sunlight exposure time relied on self-reports from participants, which may introduce recall bias and subjective assessment. Second, the observational nature of this study prevents us from establishing causality. Third, the associations between sunlight exposure and brain structure were not observed in groups with specific diseases due to the relatively small number of participants with those conditions. Fourth, the participants in this study were primarily white individuals from high-latitude regions, which may limit the generalizability of the findings to other regions and ethnicities. Fifth, given that the data on ozone and outdoor occupation status were not provided in the UK Biobank, these two potential confounding factors were not adjusted in our study. Therefore, caution is needed when interpreting and generalizing our study results.

## Conclusions

In conclusion, this study reveals an association between prolonged exposure to natural sunlight and adverse changes in brain structure, providing novel insights into the potential impact of light exposure on human health. The findings highlight the need for further in-depth investigations to elucidate the specific mechanisms and physiological foundations underlying this relationship. Understanding the intricacies of how natural sunlight affects brain structure is crucial for advancing our knowledge of the broader implications for human well-being.

## Methods

### Data sources and study design

**Figure 3:**
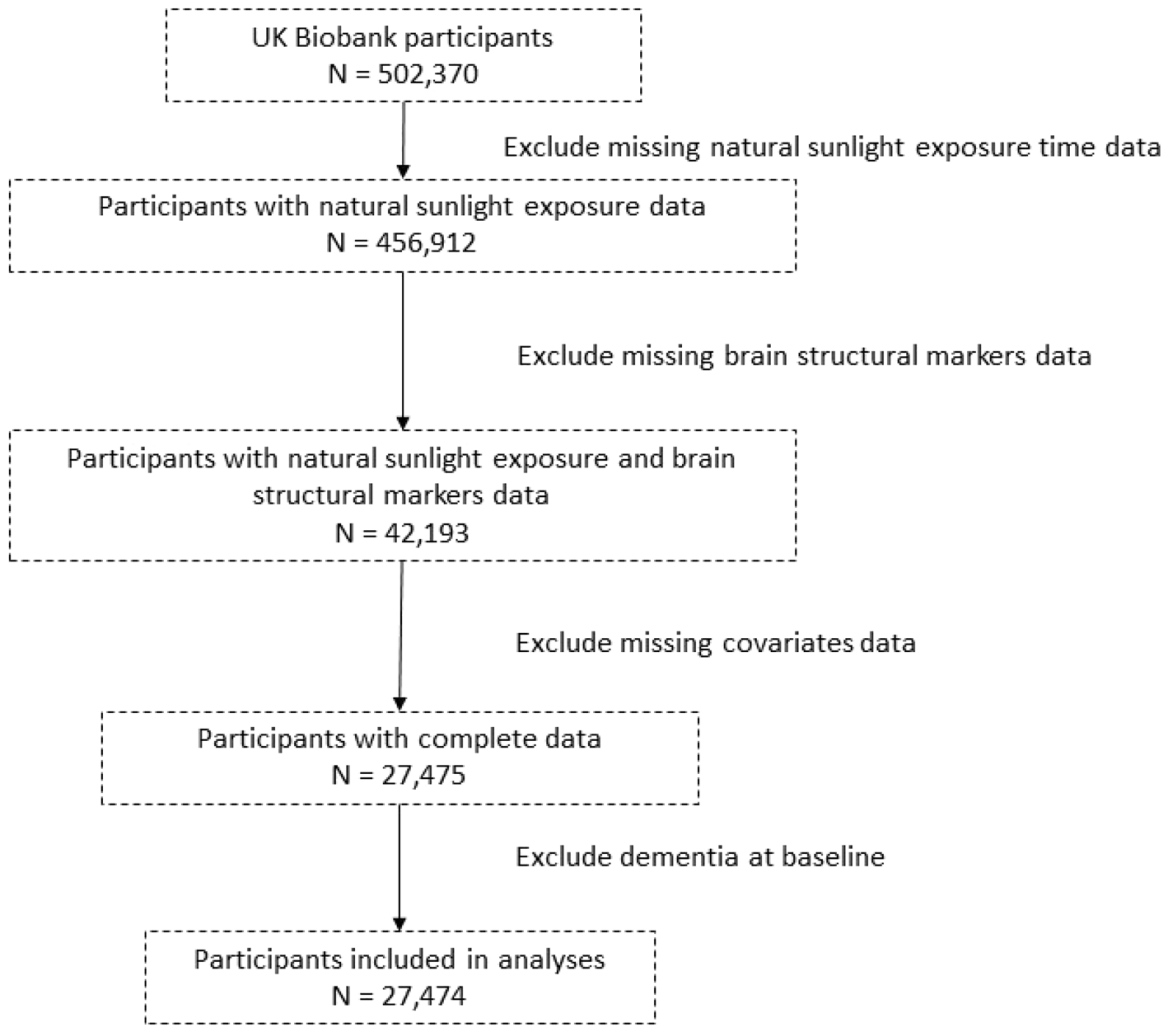
Flowchart illustrating criteria for selection of samples as well as the four analyses performed in the current study.

The UK Biobank is a population-based, large-scale prospective cohort study that recruited over 500,000 participants nationwide from March 2006 to December 2010. After signing the written informed consent forms, all participants completed baseline assessments at one of the 22 assessment centers, which were in England, Scotland, or Wales. These assessments included touchscreen questionnaires, verbal interviews, physical examinations, and the collection of biological samples. Starting in 2014, a subset of participants was invited to four assessment centers for cognitive function questionnaires, imaging scans, and more. The UK Biobank has obtained approval from the Northwest Multi-Center Research Ethics Committee (reference 06/MRE08/65). All research was performed in accordance with relevant guidelines/regulations and participants provided informed consent. The specific selection process flowchart is presented in Fig. 3.

### Natural sunlight exposure time measurements

The time spend in summer and winter was collected through touchscreen questionnaires during participants’ visits to the assessment center from 2006 to 2010. Responses of “Don't know” and “Prefer not to answer” are excluded, and “Less than 1 h” was redefined as 0 h. Participants who reported the time exceeding 16 h in summer and 8 h in winter were removed based on the effective daylight hours in the UK. The exposure variable was the annual average sunlight exposure time, which was calculated by taking the average outdoor time during both the summer and winter.

### Brain structural markers measurements

The brain structural markers including the volumes of total brain, white matter, gray matter, and white matter hyperintensities had been collected since 2014. We performed Z-transformations on the brain structural markers based on the mean and standard deviation. T1-weighted data was acquired on a 3T Siemens Skyra scanner using a standard 32-channel head coil. The parameters for the magnetization-prepared rapid gradient-echo imaging sequence were set as follows: resolution: 1 × 1 × 1 mm, feld-of-view (FOV): 208 × 256 × 256 matrix, duration: 5 min. Subcortical structures were segmented using FIRST (version 5.0), an integrated registration and segmentation tool within FMRIB. Cortical tissue-type segmentation was completed using FAST, FMRIB’s automated segmentation tool. The white matter hyperintensities were calculated based on T1 and T2 FLAIR. The UK Biobank team processed and quality-controlled the estimates of white matter volume, providing them as image-derived phenotypes to approved researchers.

### Covariates

Based on prior studies on sunlight and cognitive function, the following factors were identified as potential confounding variables: age, sex (male or female), Townsend Deprivation Index (TDI), years of education(10-years, 13-years, 15-years, 19-years, or 20-years)^57^, employment status (yes or no), physical activity(low, moderate, high), body mass index (BMI), smoking status (never, previous, or current), alcohol drinker status (never, previous, or current), skin color (very fair, fair, light olive, dark olive, brown, black), use of sun/UV protection (never/rarely, sometimes, most of the time, always, do not go out in sunshine), history of fractures in the past 5 years (yes or no), vitamin D supplementation (yes or no), sleep duration (7–8 h or not), history of hypertension (yes or no), history of stroke (yes or no), and history of diabetes (yes or no). The physical activity was accessed by using the International Physical Activity Questionnaire (IPAQ) and grouped based on derived MET (metabolic equivalent) scores following the guidelines of the IPAQ.

In addition, we further adjusted for PM2.5^58^ and polygenic risk score for Alzheimer’s disease (AD-PRS) to control environmental pollution factor and genetic factor. The assessment centers were adjusted to control the impact of the brain scanning device. The detailed definitions of hypertension, stroke, coronary heart disease, and diabetes can be found in Supplementary Table S1.

### Statistical analyses

Normally distributed variables were presented as mean (standard deviation), non-normally distributed variables as median (interquartile range), and categorical variables as numbers (percentages).

Participants were stratified into three groups based on the tertiles of sunlight exposure time (Tertile 1: ≤ 1.5 h, Tertile 2:1.5–3 h Tertile 3: > 3 h), with the group having the lowest sunlight exposure time (Tertile 1) considered as the reference group. General linear regression analysis was employed to compare the differences in brain structural markers among different sunlight exposure time groups. In stratified analysis, the subjects were divided into subgroups based on sex, age (< 60, ≥ 60), and disease history (hypertension, stroke, and diabetes). Within each subgroup, we analyzed the relationships between sunlight exposure time and brain structural markers. Additionally, we treated sunlight exposure time as a continuous variable and employed the “*plotRCS*” package for restricted cubic splines to examine the dose–response relationship between sunlight exposure time and brain structural markers. Given variations in daylight duration between seasons, we also separately examined the dose–response relationships in summer and winter.

In sensitivity analyses, we separately excluded participants who developed dementia in the first 5 years of follow-up and 10 years of follow-up, to control for potential reverse causality. Participants with a history of hypertension, diabetes, or stroke at baseline were excluded, and then repeating the primary analysis in a relatively healthy population. In addition, we conducted sensitivity analyses in white populations. The relationships between sunlight exposure time and different cognitive domains were also analyzed. (Supplementary Table S2)^59^.

The statistical analyses were conducted in R version 4.2.3, and statistical significance was set at the *P*-value < 0.05 for two-tailed tests.

### Preprints

This manuscript has been submitted as preprint at MedRxiv: 10.1101/2023.10.12.23296944.

### Supplementary Information

Supplementary Information.

## Abbreviations

UV: Ultraviolet
TDI: Townsend Deprivation Index
BMI: Body mass index
AD-PRS: Polygenic risk score for Alzheimer’s disease
